# A machine learning framework for rapid forecasting and history matching in unconventional reservoirs

**DOI:** 10.1038/s41598-021-01023-w

**Published:** 2021-11-05

**Authors:** Shriram Srinivasan, Daniel O’Malley, Maruti K. Mudunuru, Matthew R. Sweeney, Jeffrey D. Hyman, Satish Karra, Luke Frash, J. William Carey, Michael R. Gross, George D. Guthrie, Timothy Carr, Liwei Li, Hari S. Viswanathan

**Affiliations:** 1 Los Alamos National Laboratory, Los Alamos, NM 87544 USA; 2 Watershed & Ecosystem Science, Pacific Northwest National Laboratory, Richland, WA 99352 USA; 3 Department of Geology & Geography, West Virginia University, Morgantown, WV 26506 USA

**Keywords:** Natural gas, Computational science

## Abstract

We present a novel workflow for forecasting production in unconventional reservoirs using reduced-order models and machine-learning. Our physics-informed machine-learning workflow addresses the challenges to real-time reservoir management in unconventionals, namely the lack of data (i.e., the time-frame for which the wells have been producing), and the significant computational expense of high-fidelity modeling. We do this by applying the machine-learning paradigm of transfer learning, where we combine fast, but less accurate reduced-order models with slow, but accurate high-fidelity models. We use the Patzek model (Proc Natl Acad Sci 11:19731–19736, 10.1073/pnas.1313380110, 2013) as the reduced-order model to generate synthetic production data and supplement this data with synthetic production data obtained from high-fidelity discrete fracture network simulations of the site of interest. Our results demonstrate that training with low-fidelity models is not sufficient for accurate forecasting, but transfer learning is able to augment the knowledge and perform well once trained with the small set of results from the high-fidelity model. Such a physics-informed machine-learning (PIML) workflow, grounded in physics, is a viable candidate for real-time history matching and production forecasting in a fractured shale gas reservoir.

## Introduction

Energy extraction from conventional reservoirs involves producing crude oil, natural gas, and its condensates from rock formations that have high porosity and permeability^1^. Such rock formations are usually found below an impermeable caprock. For reservoir management, oil and natural gas industries rely on forecasting of future oil/gas production, which is accomplished through large investments in detailed characterization efforts and accurate history matching studies. These efforts are backed by the knowledge that reservoir productivity is consistent and reliable. The physics-based workflows adopted for modeling conventional reservoirs use extensive site-characterization data (which is acquired over months to years) for history-matching. These workflows employ detailed physics models, referred to as high-fidelity models in this paper, to perform simulations that are expensive to run. For example, it can take several days to months to run reservoir-scale model simulations^2–4^ with degrees-of-freedom of the order of hundreds of millions on state-of-the-art high-performance computers (HPC). In other words, these current physics-based approaches are not amenable to real-time decisions even for conventional reservoirs that are dominated by porous flow.

Unconventional reservoirs pose an even greater challenge for real-time forecasting: the physics of fluid flow is a complex combination of processes in micropores (< 2 nm) and mesopores (2–50 nm) and in comparatively larger fractures^5^. Unconventional reservoirs typically have fractured effective porosity in the range of 0.04–0.08 and fractured permeability of the order of nanodarcies ($10^{-16}$–$10^{-20}$ $\mathrm {m}^2$)^2,4^. Note that the porosity and permeability of shale are an order of magnitude less than fractured shale^6^. As a result, drilling and stimulation techniques are used to generate fracture networks to provide greater flow out of the reservoir^7^. However, reservoir flow becomes dominated by complex physics that operate on different timeframes; more importantly, it results in poor recovery efficiencies. The current efficiency of extraction from unconventional reservoirs is very low (approx. 5–10% for tight oil and about 20% for shale gas)^8,9^ compared to that of conventional reservoirs where efficiency of extraction ranges between 20 and 40%^10^.

Thus a model-based optimization for real-time forecasting in unconventional reservoirs could have significant impact on improved recovery and operational economics by providing insights into how different operational decisions will impact future production efficiency. However, the strategies and workflows used in conventional reservoirs are either inapplicable, or prohibitive in case of unconventionals. Due to the long horizontal laterals and fewer wells drilled, there is insufficient reservoir characterization data to inform high-fidelity physics models^4,11^. Moreover, history matching with high-fidelity models takes time during the early phase of a well’s production when the majority of hydrocarbons are produced, and the inherent rapid initial decline in production rates and the unpredictability of the yield in unconventionals suggests that the window of opportunity to exploit the reservoir is limited. As a result, workflows with high-fidelity simulations are not ideal for usage in comprehensive uncertainty quantification studies that require 1000s of forward model (forecasting) runs. The unpredictability is suspected to be due to the poorly understood nature of the physics of the drawdown process, which is exacerbated by the impact of resource development processes themselves (e.g., slow drawdown or fast drawdown)^2,4^. Thus innovative extraction strategies and advanced workflows could significantly improve the hydrocarbon recovery efficiency and long-term economics^12–14^.

Current workflows for unconventional reservoirs are predominantly based on production decline curve analysis and its extensions^15–17^, data-driven machine-learning (ML) approaches^11,18–26^, and/or extension of physics-based conventional reservoir workflows^2–4,27–30^. Decline curve analysis provides empirical models to forecast production data based on the past production history. However, this type of approach lacks direct consideration of the governing physical processes, with the impact of these processes instead embodied indirectly in empirical fits. The data-driven ML approaches perform poorly when faced with uncertain, redundant, missing, and sparse data—all of which are common occurences with existing datasets related to unconventional reservoirs. Moreover, the data-driven ML analyses perform poorly in making forecasts outside of their training regimes, whereas the exploration of novel production strategies fundamentally requires extrapolation (where ML struggles) as opposed to interpolation (where ML excels)^31^.

We believe that an approach based on physics-informed maching learning (PIML) can overcome these challenges and lead to new workflows that provide operators the ability to improve the recovery efficiency from fractured shales. We have developed and tested such an approach by leveraging the data collected at the Marcellus Shale Energy and Environment Laboratory (MSEEL) in West Virginia^32^. MSEEL is part of the US Department of Energy’s network of field laboratories that are focused on developing the science and technology needed to increase recovery efficiency from fractured shales across several US plays. The MSEEL site aims to provide a well-documented baseline of reservoir characterization. Access to multiple Marcellus wells separated by sufficient time to analyze data allows for the collection of samples and data, and the testing and demonstration of advanced technologies. The project’s phased approach allows for flexibility to identify and incorporate new, cost-effective technology and science focused on increasing recovery efficiency, while reducing environmental and societal impacts.

Aided by the MSEEL venture at this crucial juncture, we present an alternative workflow for unconventional reservoirs in this article, based on the interplay between reduced-order models, synthetic data and machine-learning. In order to explain further, we first clarify what we mean by “reduced-order models” and “synthetic” data. Reduced-order models are low-fidelity forward models, either physics-based or data-driven, and are constructed to be fast emulators of their high-fidelity counterparts, though not as accurate. In any reservoir, subsurface data/features are the independent variables that are thought to determine the production (dependent variable in this particular case). By data, we mean measurements and observations of bottom hole pressures from pressure gauges, production from flow meters, and dip, azimuth etc., of fractures that are sampled on site. When we say unconventionals are data sparse, we mean that these measurements/observations in themselves are not enough for prediction. By synthetic data, we mean all data that is a result of computational simulation with a physics model using realistic site-specific parameter ranges. The inputs to these models may not be completely known from the site data, except for broad ranges, so in that sense the data obtained from their outputs is synthetic. Thus, synthetic data-sets can be produced from both low-fidelity or high-fidelity models. They differ in the computational expense needed to generate them, and the degree of fidelity with reality. Synthetic data from low-fidelity models are easier to generate, but are less accurate, and vice versa for high-fidelity models. We can augment field data with synthetic data, provided that they accurately capture physical phenomena and a range of feature combinations that are representative of the site.

The motivation to generate synthetic data from both high-fidelity and low-fidelity models comes from the paradigm of transfer learning^33,34^, wherein neural networks trained with copious data from fast reduced-order models are then updated with modest amount of available data from the field or high-fidelity models. This allows us to effectively use the ML techniques that need copious amounts of data on sparsely available field or the high-fidelity data from simulations of the site, and thus circumventing the difficulties pertaining to insufficient data that plague current ML approaches to forecasting for unconventionals. We present a PIML workflow rooted in transfer learning to address the challenges to real-time reservoir management in unconventionals. We believe such a PIML workflow, grounded in physics, is a viable candidate for real-time history matching and production forecasting in a fractured shale gas reservoir. We note that training an ML model with multi-fidelity data is not new^35–37^. However, the use of transfer learning in the context of real-time workflows for unconventional reservoir management is new, and that is what we present as our novel contribution.

Since transfer learning and PIML are interpreted in multiple ways, we now clarify why and how our approach is in keeping with the definitions. Transfer learning is a research problem in machine-learning (ML) that focuses on storing knowledge gained while solving one problem and applying it to a different but related problem. In more concrete terms, given a source domain $D_s$ and learning task $T_s$, a target domain $D_t$ and target learning task $T_t$, where $D_s \ne D_t$ or $T_s \ne T_t$, transfer learning aims to improve the learning of the target predictive function in $D_t$ using the knowledge in $D_s$ and $T_s$. In our context, the first problem is the inverse of a reduced-order model, and the second is the inverse of the full model. The use of transfer learning makes sense in this context because there is not sufficient data from the full model that can be used to train the target predictive function. We also emphasize that our workflow is not a regular application of an artificial neural network model, since we are using a physics model within the ML workflow. Making a learning algorithm physics-informed^38^ amounts to introducing appropriate observational, inductive or learning biases that can steer the learning process towards identifying physically consistent solutions. In this process, observational biases can be introduced directly through data that embody the underlying physics (which is what we have done in this article) so that training an ML model on such data allows it to learn functions that reflect the physical structure of the data.

The significance of our approach is that though it is developed for MSEEL, it can be extended to other plays. The transfer learning approach is attractive for tasks where reusability of ML-models is of great importance. We note that the same workflow can be applied to other formations (e.g., Woodford, Barnett, Utica, EagleFord) should site data become available, and the same set of ML techniques from transfer learning will be able to model another site/formation. In other words, we may be able to pre-train an ML algorithm with “generally applicable” synthetic data over a range of feature combinations. Then we can transfer the learning to a specific site rapidly using a combination of site-specific synthetic data and field data. Thus, the developed ML models for one site (e.g., MSEEL) might require fine-tuning (or minimal retraining of the neural networks) to transfer knowledge across shale sites/formations. This is not burdensome when compared to developing a new ML model for a different site altogether, making our PIML approach an attractive workflow for unconventional real-time montoring. In the subsequent sections, we first describe the new PIML workflow we propose, and demonstrate its use by predicting long-term production from knowledge of short-term production history.

## A physics-informed machine-learning approach

**Figure 1:**
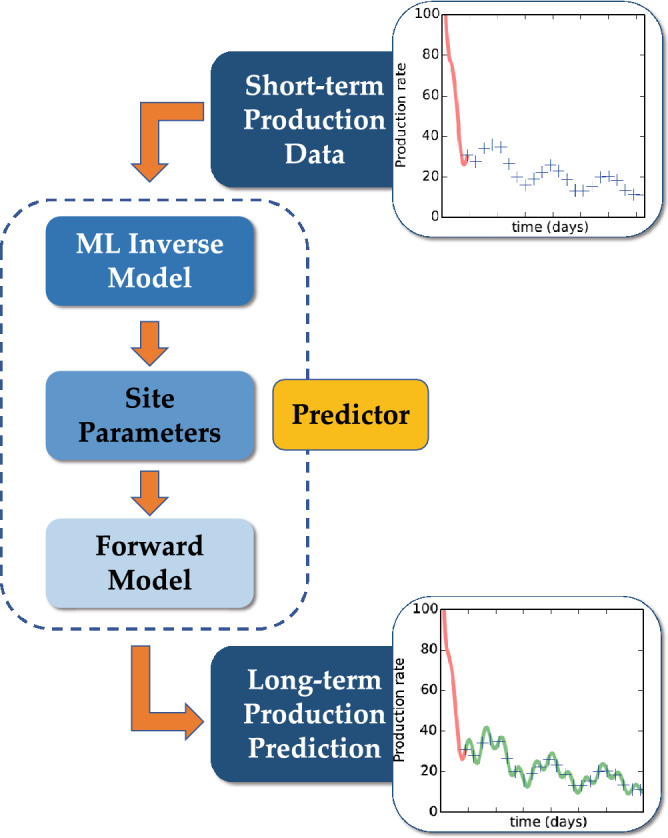
PIML workflow for reservoir management. Short-term production data is supplied to the machine-learning inverse model which predicts appropriate site parameters that are then used as inputs to the forward model. Note that the forward model could be either physics based or again a machine-learning model.

The fundamental problem of forecasting is to predict long-term production of a site, given its short-term production. The computations involved in the prediction need to be accelerated dramatically in order to achieve the goal of near real-time forecasting. For all forecasting, one needs an inverse model that can take in short-term production and infer site parameters or inputs which in turn are passed onto a forward model (forecasting model) to get long-term production. This process is illustrated by the block diagram in Fig. 1. Naturally, both components (inverse model and forward model) need to be computationally efficient to be useful in forecasting. The requirement of computational efficiency rules out a high-fidelity physics model being chosen as a forward model or as the underlying inverse model. Thus, our choices for both components are limited to either data-driven (ML) models, or reduced-order models based on simplified physics-based assumptions. For example, to model flow and transport in a three-dimensional fractured system, one can assume that the transport into the matrix follows a one-dimensional diffusion model, and the resulting fluxes can be (or subtracted) to the mass balance of the species being transported in a fracture. While reduced-order models are fast by design, ML models are fast once they are trained and calibrated.

One challenge encountered in choice of a reduced-order models occurs due to the fact that there are three primary flow regimes governing basic production behavior of a shale well^39^:

1. *Early-time linear flow regime* when flow is linear and orthogonal to the individual hydraulic fractures
2. *Transition from linear flow regime to SRV (stimulated reservoir volume) flow regime* when the fractures begin to interfere, and production begins to access the rock volume where natural fractures have been stimulated by the hydraulic fracturing process
3. *SRV flow regime* when the fractures are fully interfering, the drainage volume has accessed the entire SRV and the behavior of the well becomes similar to that of conventional wells draining from a closed volume and undergoing pseudo-state steady-state behavior.

We note that these macro-scale responses subsume a whole host of multi-scale, multi-physics processes (ranging from Knudsen flow to transition flow to slip flow to continuum flow, as indicated in^39^). The differing nature of well flow corresponding to these regimes results in production rate varying inversely as the square root of time in the linear flow regime whereas production rate follows an exponential decline in the SRV flow regime. This variation makes the problem of extrapolating early time production data to late-time estimated ultimate recovery extremely challenging for machine-learning driven workflows, which cannot extrapolate across flow regimes with changing physics.

We believe that capturing the transition flow regime will be at odds with the stated purpose to have a fast and efficient tool for forecasting. Thus, our choice of reduced-order model is predicated on capturing the early and late time behaviour described above, and the Patzek model^6^ is shown to have the right scaling with time for both regimes.

Hence, we use a reduced-order model based on the model proposed by Patzek^6^ as the forward model, and use ML to construct an inverse model. The ML inverse model takes the form of an artificial neural net (ANN) that can predict the input parameters for the forward model (reduced-order model) based on short-term production data. The fast reduced-order forward model will then use these input parameters to forecast long-term production. Since the reduced-order forward model is computationally inexpensive, one can run thousands of realizations or scenarios, fairly quickly, thus giving estimates on other decision-making quantities of interest such as uncertainty bounds, or can be used in an optimization loop to optimize economic quantities, for instance.

The challenges in designing an ML inverse model in this approach for unconventional reservoirs stem from three different aspects:

1. ML approaches require copious amounts of data. Having mentioned that unconventionals, in general, are data sparse, we must then create synthetic data-sets from physics-based models.
2. Using high-fidelity physics models for generating data-sets is infeasible since they are computationally intensive.
3. Using data-sets generated by low-fidelity physics models alone is also infeasible because without the inputs from high-fidelity models, ML approaches will not be trained on the full physics.

**Figure 2:**
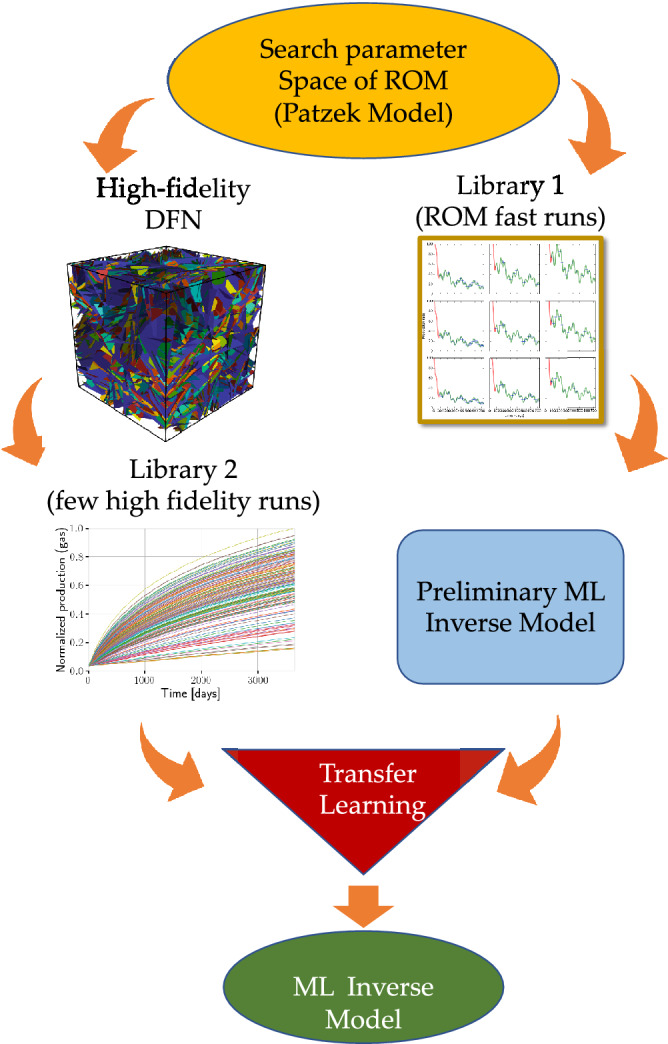
A magnified look at the steps to produce an ML inverse model illustrating how the transfer learning paradigm uses both synthetic data from reduced-order models as well as high-fidelity models. Note that in this workflow, we used Patzek model as the reduced-order model, but the same workflow can be used with other reduced-order model choices that may be physics-based or even data-driven.

Thus we are forced to look for an approach that uses both high-fidelity models and reduced-order models in tandem with machine-learning. The steps involved in construction of an ML inverse model are described in Fig. 2. In such an approach, a large number of simulations with reduced-order models will allow for the generation of synthetic data over a broad range of potential site conditions for training machine-learning models. Once site data become available, subsequent training focusses on a smaller set of synthetic data generated for specific site conditions. In other words, the final training done using a limited number of simulations with high-fidelity models will incorporate the physical insights that were missing in the initial training with data from the reduced-order model, thus circumventing the three challenges enumerated earlier. This approach, known as Transfer Learning^34^, is shown in Fig. 2 and will be described in greater detail after a brief description of the high-fidelity model and the reduced-order model used in this study.

### The high-fidelity DFN model

**Figure 3:**
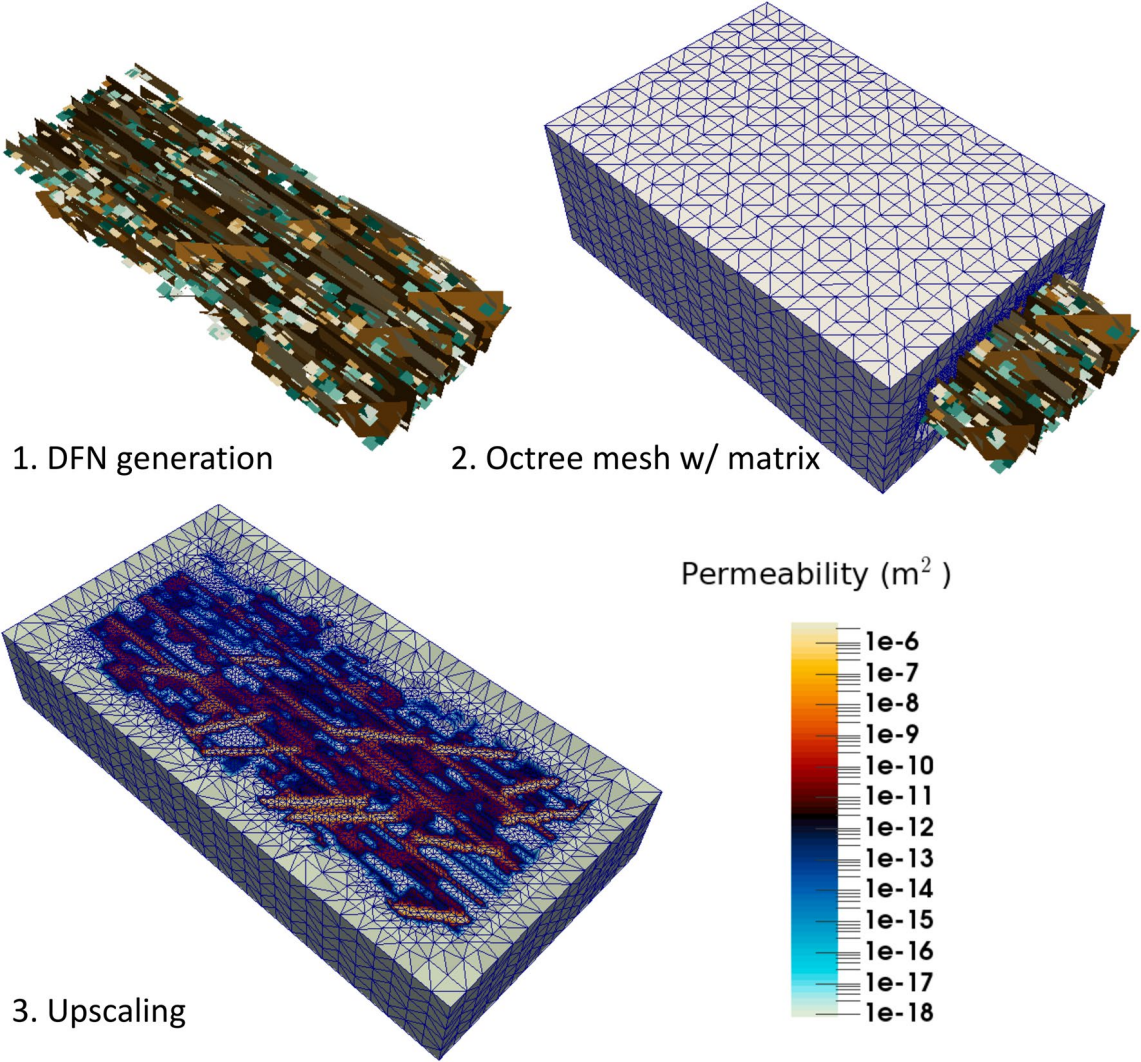
The DFN model with an octree-refined continuum mesh has $5\times 10^{5}$ cells, despite using upscaling for permeability and porosity. There is large contrast in permeability and porosity since fractures are more permeable than the matrix, resulting in a stiff system after numerical discretization. Thus, it is difficult to simulate gas flow and transport for long periods of time, rendering the approach infeasible as a tool to generate large datasets or as a fast forward model for forecasting.

A discrete fracture network (DFN) model of a single stage was constructed based on field data from the MIP-3H well at the MSEEL site (see Fig. 3). DFN models represent fractures as two-dimensional planes in three-dimensional space and are widely used for modeling flow and transport in fractured reservoirs, e.g.^40^, and references therein. DFNs allow for accurate representations of unconventional reservoirs because fracture geometries and characteristics gleaned from field data can be explicitly accounted for in the model. However, DFNs do not account for the surrounding matrix, so we take advantage of recent developments in continuum modeling to accurately capture both the fracture and matrix effects needed to model unconventional reservoir production.

The fracture data were collected from a 34 m slabbed core and include measurements of the number and lengths of different types of fractures. In general, there are three types of fractures that were included in the DFN: vertical opening-mode fractures (strike and dip of: 261, 84N), horizontal opening-mode fractures (strike and dip of: 037, 01SE), and two sets of faults (i.e. shear fractures; strike and dip of: 048, 34SE and 211, 45NW). The fracture orientations were assumed to follow a Fisher distribution and the $\kappa$ values (i.e. a measure of the clustering) for each fracture type were calculated as: 16.0, 1148.5, 43.0, and 98.87, respectively. The lengths for each fracture type were measured directly from core exposure and the apertures were assumed to be length correlated. The hydraulic fractures are part of the original DFN and we do not model hydraulic fracture stimulation or growth. In this particular single stage of MIP-3H, we used micro-seismic data to constrain the height and lengths of the 3 hydraulic fractures. Lastly, we model the well as a single fracture orthogonal to the three hydraulic fractures, which is used as a boundary condition in the simulations.

We built a high-fidelity DFN based on the field data using dfnworks^41^. The DFN includes 3 hydraulic fractures and 1230 natural fractures. Based on the DFN, we generated a three-dimensional octree-refined continuum mesh to capture the matrix effects and upscaled the fracture network to generature effective permeability ($10^{-6}$ Da) and porosity (0.075) values that are needed to simulate gas flow and transport^42^. To illustrate why it is infeasible to use high-fidelity models alone for generation of data sets or prediction, we note that the final mesh contains approximately half a million cells, so the large number of cells combined with the low permeability of the matrix makes the problem numerically stiff, taking wall-clock times of the order of hours for simulation even with parallelization.

**Figure 4:**
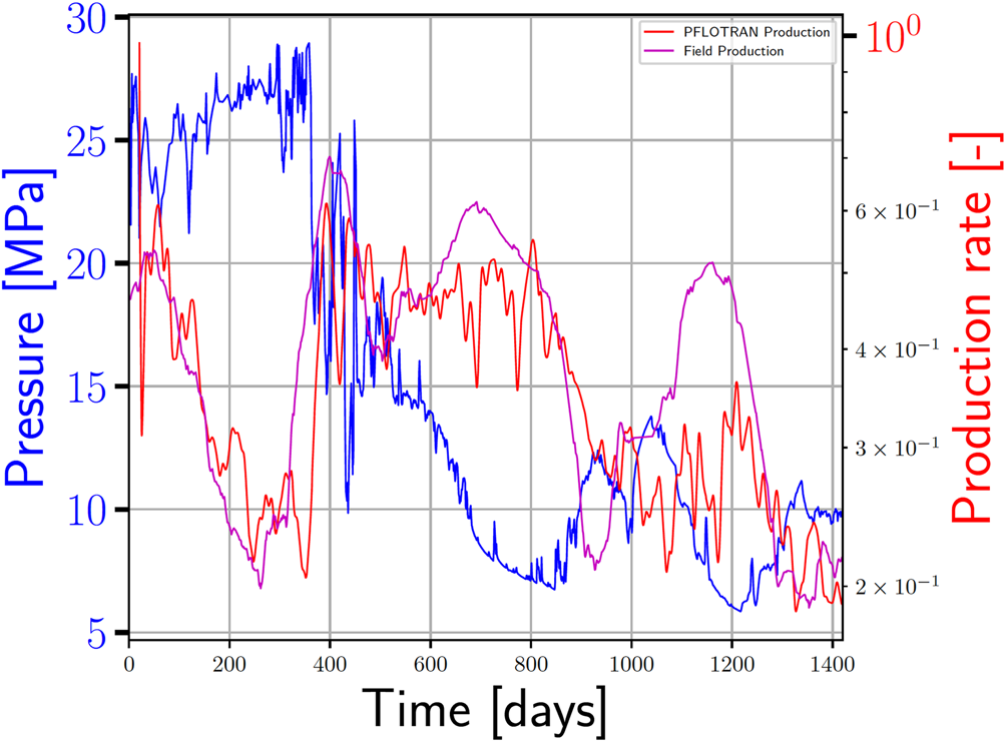
Benchmarking our model using actual dynamic production data from the MIP-3H well at the MSEEL site. The well boundary is assigned the same pressure as the well at the site (blue line) and we measure the amount of gas that is removed from the system. The agreement between the production from our simulated system (red line) agrees quite well with the field data (magenta line). Some differences are to be expected due to the assumptions in the model, but they do not appear to affect the first order behavior, especially for the first few years of production. At later times, the model diverges slightly, which could be due to second order effects (e.g., fracture mechanics and stimulation, interacting wells) that are not included in the model.

The full physics model used to simulate gas flow and transport in fractured porous media (Sect. 2.4 in Ref.^43^) assumes single-phase, isothermal gas flow and transport using properties of methane as the single gas species. The modeling assumptions also include using Darcy’s law and Fick’s law for flow and transport, respectively^2,4,44^. Use of a single-phase model is appropriate since there is negligible quantity of water or oil at this site. However, adsorption and nano-pore confinement effects on phase behaviour are ignored. In addition, we do not consider the effects of matrix permeability changing due to stimulated natural fractures. We use pflotran^45^, a state-of-the-art simulator of flow and transport through the DFN model and develop high-fidelity simulation data. pflotran uses a two-point flux finite volume discretization with a backward Euler scheme for time-stepping and a Newton-Krylov solver to solve the system of nonlinear equations that arise after discretization.

We benchmark our model using actual dynamic production data from the MIP-3H well at the MSEEL site (Fig. 4). We assign the well boundary condition the same pressure as the well at the site (blue line) and measure the amount of gas that is removed from the system. As can be seen in Fig. 4, the agreement between the production from our simulated system (red line) agrees quite well with the field data (magenta line). We would expect some differences due to the assumptions in the model, but they do not appear to affect the first order behavior, especially for the first few years of production. At later times, the model diverges slightly, which could be due to second order effects (e.g., fracture mechanics and stimulation, interacting wells) that are not included in the model. Nonetheless, the agreement is sufficient to provide scenarios for the transfer learning.

### Reduced-order models for fractured reservoirs

For fractured reservoirs, there exist different classes of reduced-order models, derived from different fundamental starting points. If matrix diffusion effects may be deemed unimportant, and the high-fidelity model is represented by the simulation of advective transport through a DFN, then there exists a family of reduced-order models whose members represent different levels of fidelity and are derived from distinct assumptions. There are graph-based reduced-order models based on mapping a DFN to graph^46^, and reduced DFNs themselves obtained by use of graph-theory or machine-learning or a combination of both^47^.

However, the model proposed by Patzek et al.^6^ is the simplest model (spatially one-dimensional time-dependent partial differential equation) of gas production consistent with the basic physics and geometry of the extraction process, and we choose it to be the low-fidelity model in our PIML workflow. Patzek et al. state that while the solutions of the model depend upon many parameters in theory, in practice and within a given gas field, all but two can be fixed at typical values, leading to a nonlinear diffusion problem solved exactly with a scaling curve. This simple model provides a surprisingly accurate description of gas extraction from 8294 wells in the United States’ oldest shale play, the Barnett Shale^6^ and it has also been applied to the Marcellus wells^48^.

Since MSEEL is a gas-dominated play, concentration and pressure both follow the same diffusion-type PDE, and we consider a simplified form of the Patzek model governing the gas concentration *c* (dimension $ML^{-3}$) in Eqs. (1)–(6), where *x*, *t* denote the space and time variables respectively. The two site-dependent parameters $\alpha$ (dimension $L^2T^{-1}$) and *m* (dimension $ML^{-3}$) that appear in the model act as the upscaled effect of the permeability, diffusivity, fracture geometry and the amount of hydrocarbon in place.

1 $$\begin{aligned}&\dfrac{\partial c}{\partial t} = \alpha \dfrac{\partial ^2 c}{\partial x^2}, \end{aligned}$$

2 $$\begin{aligned}&c(x, t=0) = m, \end{aligned}$$

3 $$\begin{aligned}&c(x=0, t) = 0, \end{aligned}$$

4 $$\begin{aligned}&\dfrac{\partial c}{\partial x}(x=1, t) = 0, \end{aligned}$$

5 $$\begin{aligned}&\mathrm {prod}(t) = \dfrac{\partial c}{\partial x}(x=0, t), \end{aligned}$$

6 $$\begin{aligned}&\mathrm {cumprod}(t) = \int _{0}^{t}\mathrm {prod}(s)ds. \end{aligned}$$

Thus, our premise is that if the appropriate model parameters could be ‘inverted’ from the production data (synthetic or site-specific) by using a ML inverse model, as highlighted in Fig. 1 then the simplified Patzek model will serve as an effective reduced-order model for forecasting. The sequence of steps using this premise is described in Fig. 1.

The problem of identifiability of parameters affecting long-term behavior from short term production data has bedeviled unconventional reservoir engineers for quite some time^39,49^ and is also recognized by Patzek et al.^6^, who state that only the onset of interference between adjacent hydrofractures makes it possible to predict both parameters simulateneously. When we say we will observe short term production and predict long term production, we do not intend to claim that we can observe the early inverse square root decline and use that to predict future production indefinitely. We simply mean that we will observe the available data and make a prediction that is accurate for some time into the future. One of our goals working with data from the MSEEL site is to help characterize the accuracy of the predictions given various observation windows at various times into the future.

### The ML inverse model

**Figure 5:**
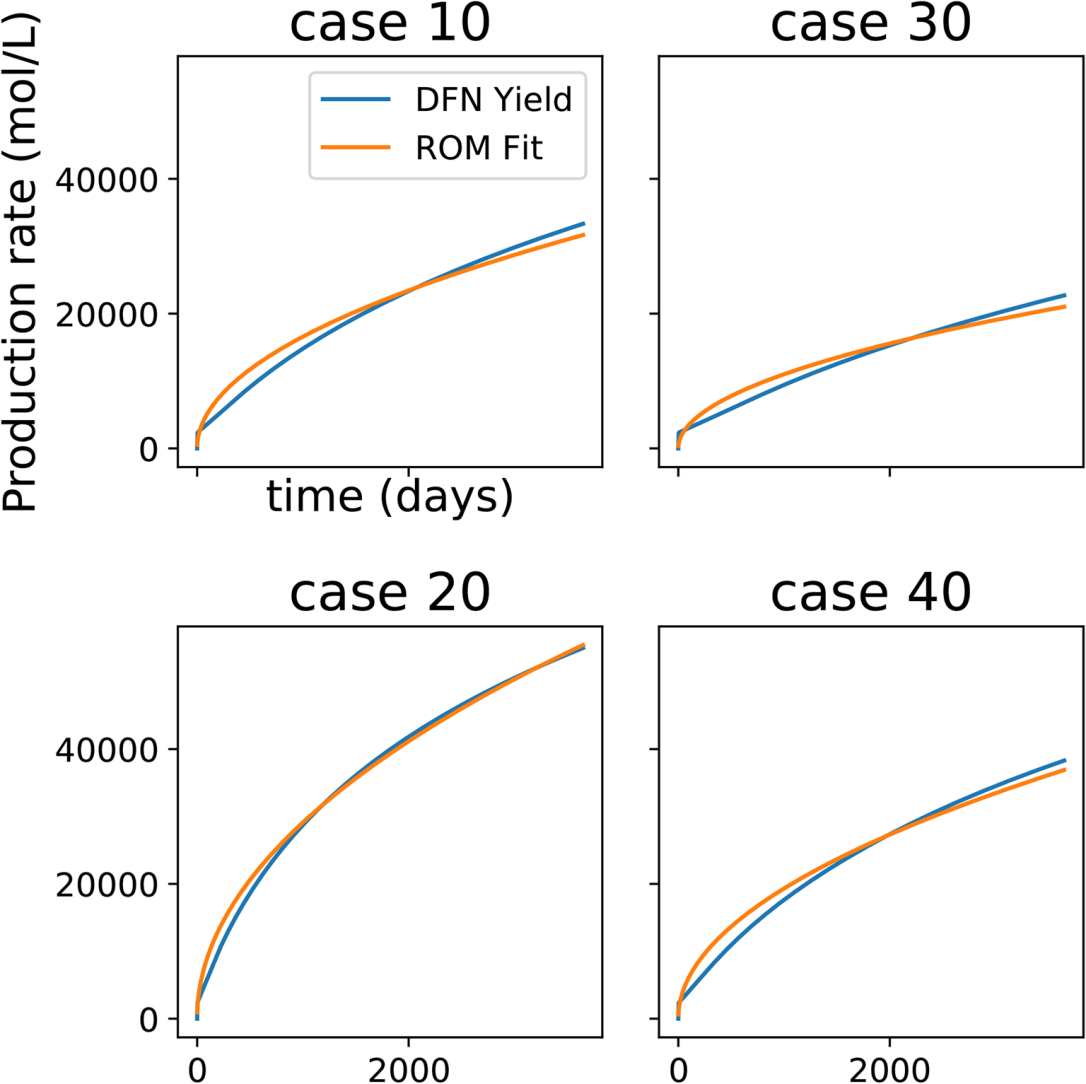
The Patzek reduced-order model can accurately fit the production data obtained from the high-fidelity model runs. This is evident from the root mean square (RMS) error of the fit, which is less than $10^{-5}$ in each of these plots.

Here, we describe the methodology to construct the ML inverse model in Fig. 2. The ML inverse model takes the form of an artificial neural net (ANN) that can predict the site parameters ($\alpha , m$), based on short-term production data. What this means is that the input to this ANN (or the ML inverse model) is the short-term production data and the output are the parameters $\alpha , m$. Now, to train the ANN, we need production data labelled with the true values of the parameters. For this purpose, we first use a handful of the DFN high-fidelity simulations, and then determine the approximate range within which the parameters $\alpha , m$ lie. The one-to-one correspondence between the production data from the high-fidelity model and model parameters $(\alpha , M)$ for the low-fidelity model comes from curve fitting with a minimum norm least squares method. From the curve fitting exercise, we verified that the Patzek reduced-order model can be fitted to show excellent agreement with the production curves obtained from the high-fidelity model runs (see Fig. 5). One of the goals of our workflow is to make it applicable to a variety of high-fidelity models, which requires a separation between the input parameters of the high-fidelity and low-fidelity models. This fitting procedure enables us to establish a connection between the two models. Then, we sample these two parameters out of this parameter space range and run the forward reduced-order model; thus, allowing us to create copious training data in the form of the first 90 days of production. At this juncture, a question may well arise that if the Patzek reduced-order model can accurately fit the (long-term) production data obtained from the high-fidelity model, then why use the multi-fidelity models in the first place. To answer that, we note that if all we needed was to history match, we could indeed use low-fidelity models and attain our ends. However, performing a history match of a low-fidelity model and then predicting with that will not give the best predictive performance. This requires the low-fidelity model to accurately fit the history of production and the future production—no small task. The machine-learning inverse model is trained to enable the low-fidelity model to provide the best predictive performance, even if it requires sacrificing the history-matching quality of the low-fidelity model. Nevertheless, high-fidelity models with good predictive capability exist, and we want our emulators to mimic them. Since a physics based model is mechanistic, we can calibrate the low fidelity model to high fidelity data to improve the forecasting ability of low fidelity model. Training on data from the high-fidelity model enables the combination of the ML inverse model with the low-fidelity physics model to produce predictions with quality similar to predictions from a high-fidelity model.

The training phase of the ANN is an optimization problem wherein appropriate choices of layer parameters are made to minimize a user-defined *loss function*. Let us suppose there are *n* samples in the training dataset. Let $\alpha , m$ be the true values of the Patzek parameters while $\alpha ^{p}, m^{p}$ denote the values predicted by the ML inverse model. The production data is provided for the first *S* days, and we are interested in production for the next *L* days, i.e., days $S+1, S+2, \dotsc S+L$.

Then the parameter loss function measures the deviation of the predicted parameters from the true parameters as

7 $$\begin{aligned} l = (\alpha - \alpha ^p)^2 + (m - m^p)^2. \end{aligned}$$

On the other hand, we have the true future production for *L* days as $(tp_{S+1}, tp_{S+2}, \dotsc tp_{S+L})$ while the predicted parameters $\alpha ^p, m^p$ predict a production rate $(p_{S+1}, p_{S+2}, \dotsc p_{S+L})$ from the Patzek model. Thus, we can formulate an alternative loss function that measures the error in the prediction of long-term production as follows:

8 $$\begin{aligned} l = \sum _{i=1}^{L} (tp_{S+i} - p_{S+i})^2. \end{aligned}$$

We use both loss functions in this study, but the results obtained are very similar.

We constructed a dense ANN with 4 layers (dimensions $90 \times 64, 64 \times 128, 128 \times 16, 16 \times 2$) that uses a rectified linear unit (ReLU) activation function. The input to the ANN must be a vector of length $S=90$, corresponding to short-term production data for the first *S* days, and the output from the ANN is a tuple of two numbers which represent the Patzek model parameters that are predicted to correspond to the given production data. The number of epochs was set to 50, and the ADAGrad optimizer was chosen^50^. The layer dimensions were tuned for best results before finalizing them by examining the performance of the model on a test data set to ensure that both underfitting and overfitting are avoided. Once we have a trained ML inverse model (i.e, the ANN) ready, it is used in conjunction with the forward model. Supplying short-term production data to the ML inverse model gives us approximate reduced-order model parameters, which are then used by the forward model to predict long-term production.

## Transfer learning

The advantage of using synthetic data generated by a reduced-order model is that it allows us to have data-sets of sizes appropriate for training the ANN. However, an ANN trained on large synthetic data-sets from low-fidelity models cannot be expected to generalize reliably when given real data from the site or synthetic data from high-fidelity models. Now, this is where we take advantage of the Transfer Learning paradigm, which is at the core of our PIML workflow. In this paradigm, an ANN trained on synthetic data from low-fidelity models is retrained with synthetic data from high-fidelity simulations, or whatever real data is available from experiments/measurements. In this process, the ANN weights are perturbed from their values obtained by training on data from low-fidelity models, and the transfer learning allows the determination of weights that generalize better to real data (or synthetic data from high-fidelity models). Note that we implicitly treat synthetic data from high-fidelity models as the ground truth in our transfer learning workflow. This allows us to have the speed of the low-fidelity model while retaining some of the quality of the high-fidelity model, which is never perfect but is an improvement over the low-fidelity model.

In this instance, we used $10^4$ samples generated by the Patzek reduced-order model to train the ANN-based inverse model in the first stage. In the second stage with transfer learning, we used 150 samples obtained from simulations of flow and transport through the high-fidelity DFN models.

## Results

We shall present the results of the transfer learning paradigm after providing some more details about the data-sets that were used to train and test the workflow.

**Figure 6:**
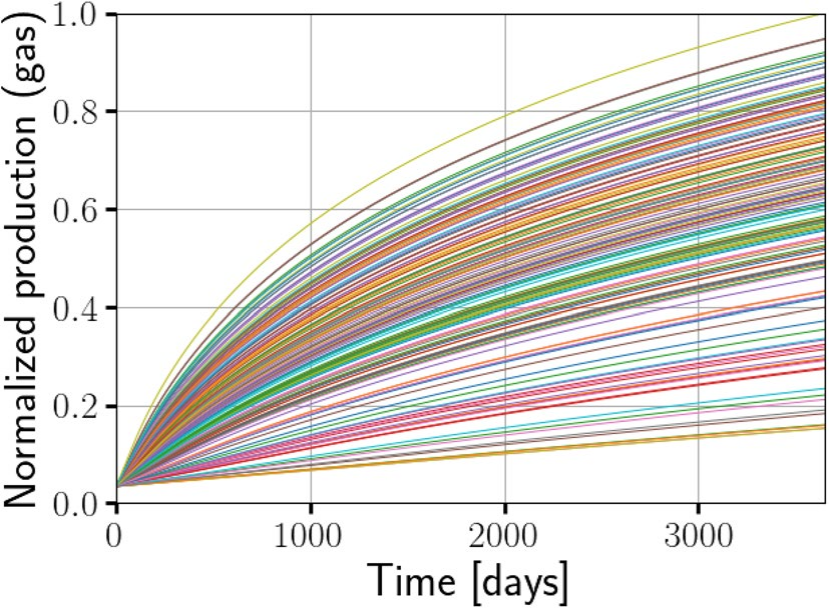
The normalized cumulative production obtained from 150 high-fidelity simulations for a time-span of 10 years is shown. The production curves show an appreciable spread corresponding to the variance in the data. The simulations correspond to 150 samples of various parameters determined by the model of the fracture network. The fracture network model is based on data from the MSEEL site.

**Figure 7:**
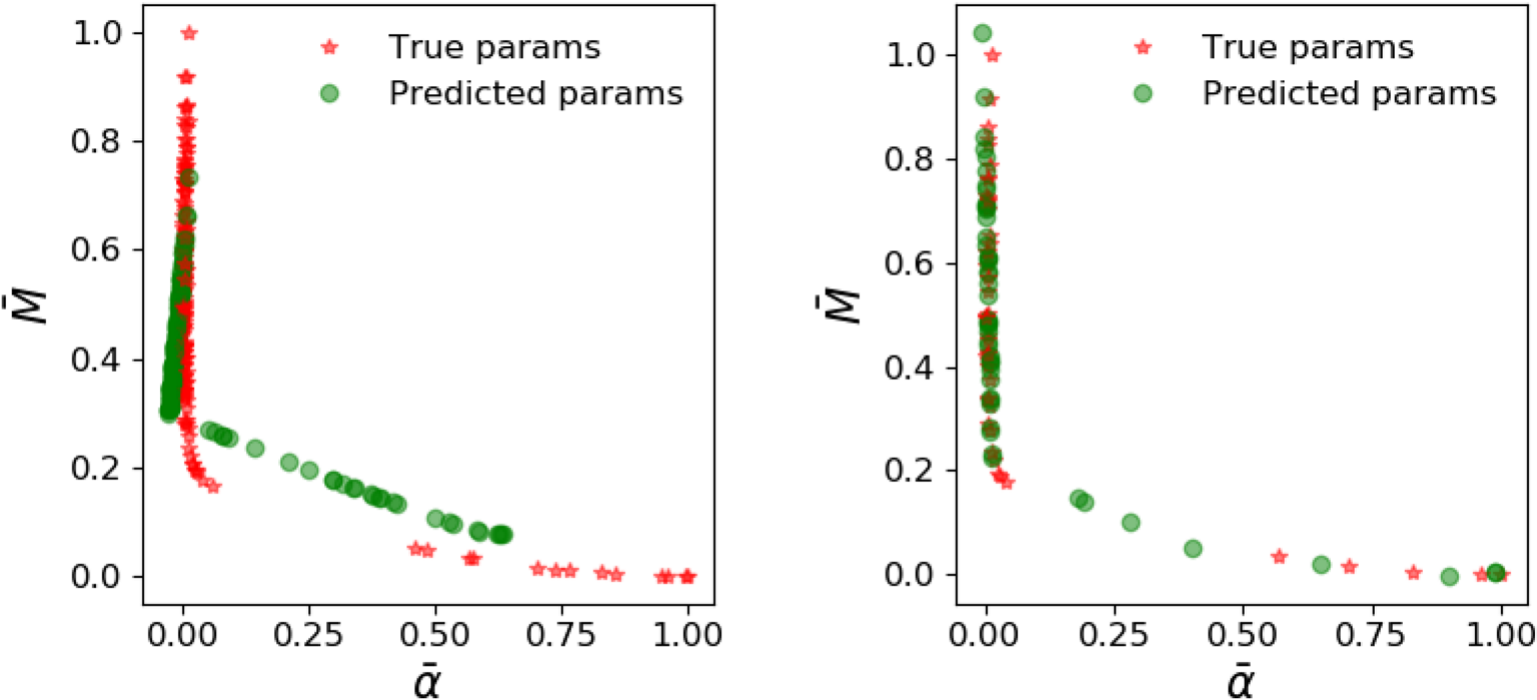
Parameter prediction from the ANN with parameter loss function (7) before (left) and after (right) transfer learning. Observe that training with synthetic data from the Patzek reduced-order model yields reasonable performance which improves remarkably after transfer learning. The distribution of the true parameters has a mean and standard deviation of 0.07 and 0.23 for $\bar{\alpha }$ and 0.49 and 0.22 for $\bar{M}$.

In Fig. 6, the synthetic production data generated from 150 high-fidelity simulations is shown. We emphasize that this synthetic production data is the end result of a time-consuming process that starts with using statistical data on fractures from the MSEEL site to build a DFN model, and then simulating flow and transport on the DFN models to obtain the predicted production data.

**Figure 8:**
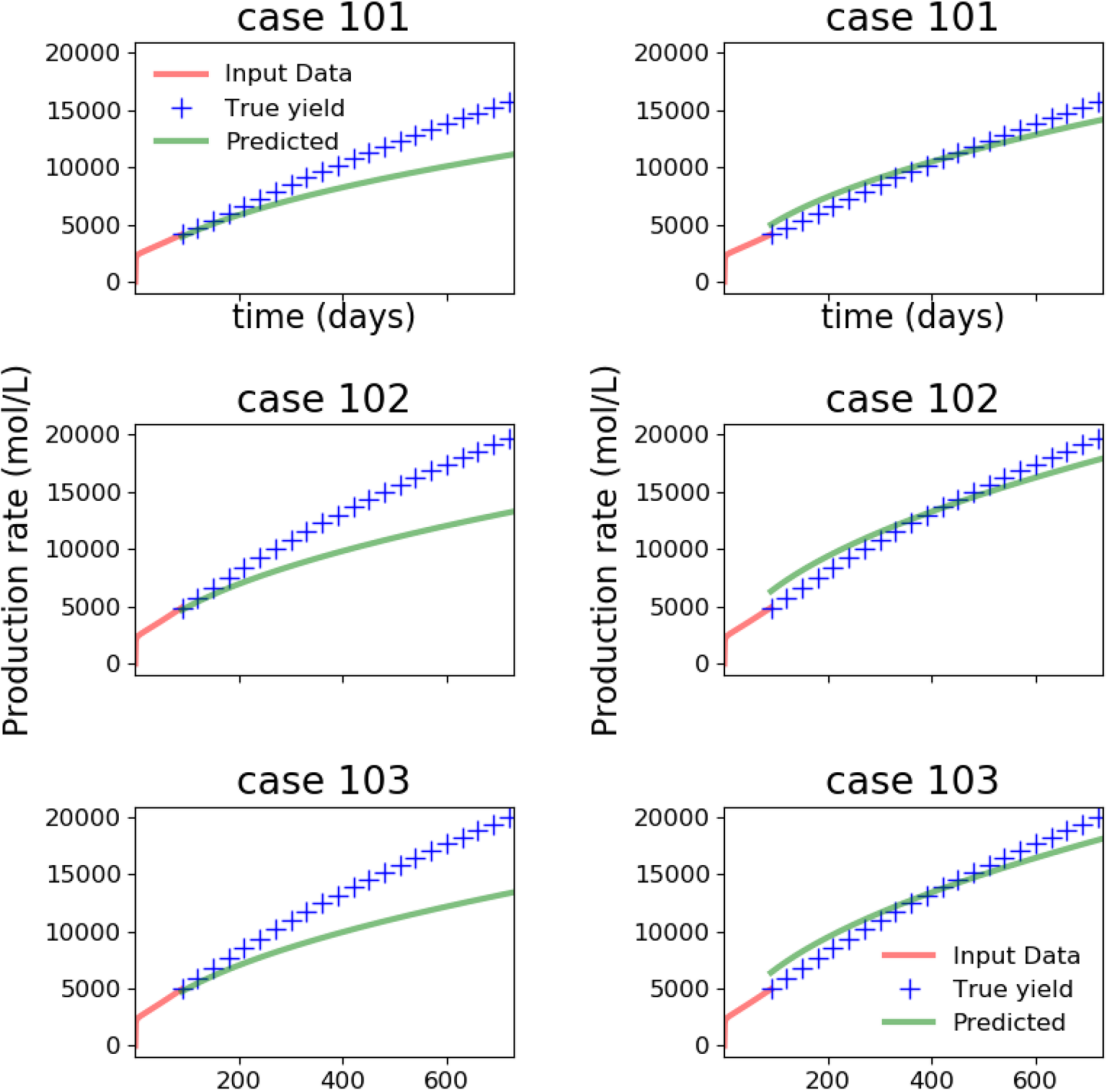
Predicted production profile obtained with Patzek reduced-order model from the ANN with parameter loss function (7) before (left) and after (right) transfer learning. In the previous figure, we observed that before transfer learning, the predicted parameters were reasonable. However, here we see that training with synthetic data from the Patzek reduced-order model is not able to capture the production profile generated by a high-fidelity model, but transfer learning with synthetic data from 100 DFN simulations is able to correct the discrepancy.

**Figure 9:**
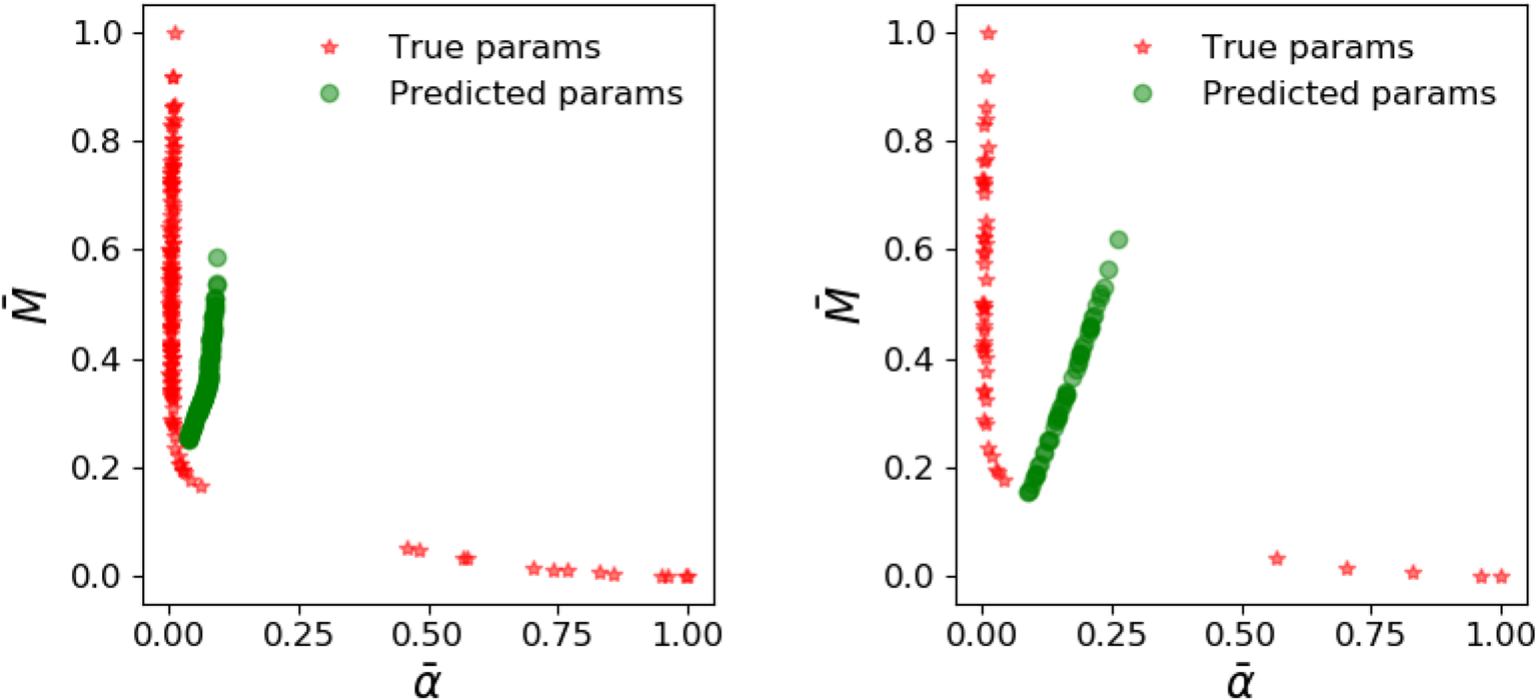
Parameter prediction from the ANN with production loss function (8) before (left) and after (right) transfer learning. The training with synthetic data from the Patzek reduced-order model is not geared to predict the parameters accurately, but instead to match the production.

**Figure 10:**
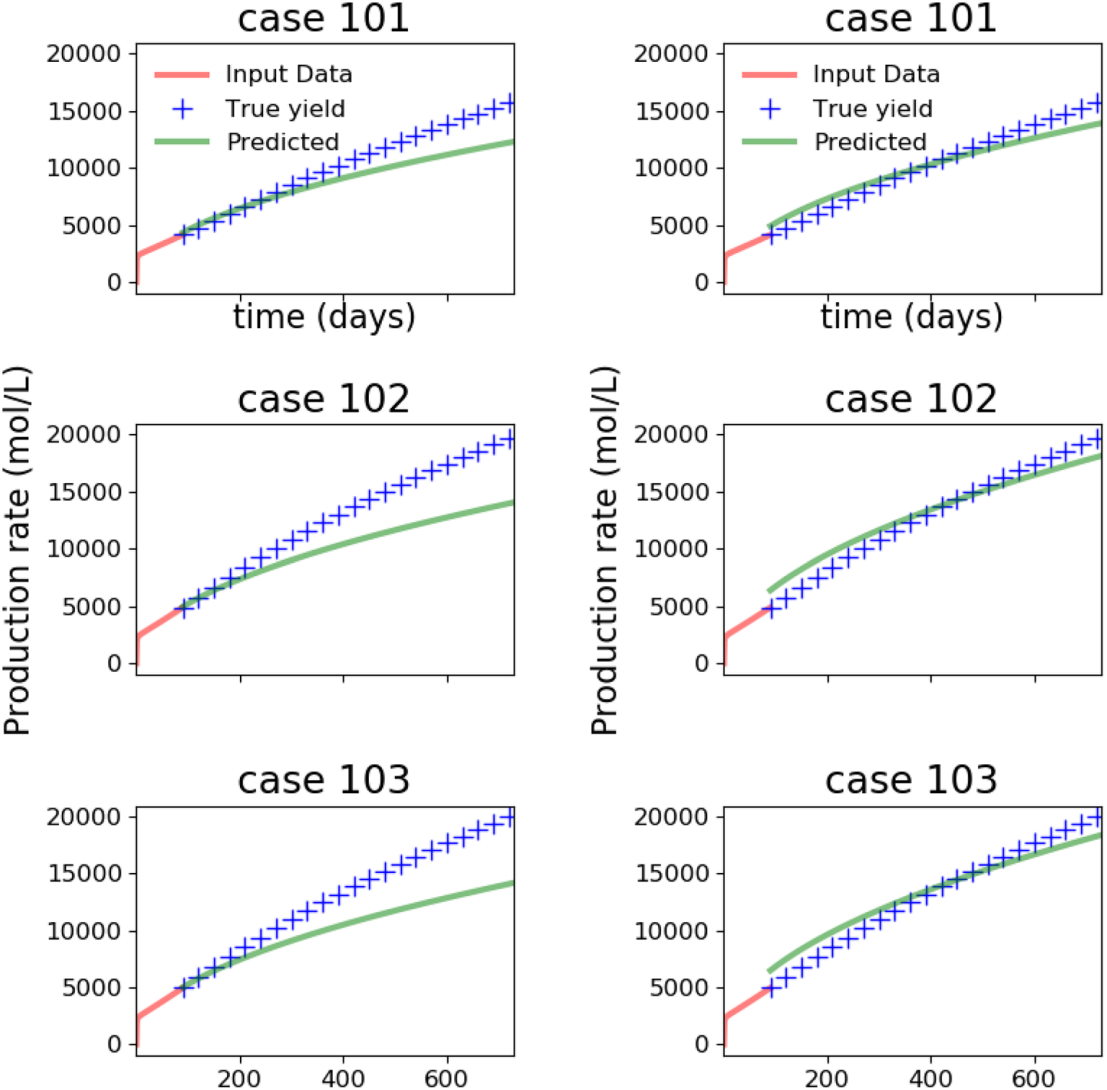
Predicted production profile obtained with Patzek reduced-order model from the ANN with parameter loss function (8) before (left) and after (right) transfer learning with data from 100 DFN simulations. Observe that training with small amount of high-fidelity data from DFN simulations allows the ANN to capture the production profile generated by a high-fidelity model, but without transfer learning, the predicted results are not useful.

However, the input parameters for the high-fidelity model are different from that required for the Patzek model (the chosen reduced-order model). Thus, we need to determine what is the range of the input parameters of the reduced-order model that will approximate the synthetic data in Fig. 6. By performing curve-fitting, we find that the Patzek parameter $\alpha$ has an order of magnitude of $10^{-5}$ but *m* has an order of magnitude that ranges between $10^3$ and $10^5$. When used as is, such values lead to ANNs that are badly-scaled, and hence we normalize the data in the following way before using:

We define scaled values $0 \le \bar{\alpha }, \bar{M} \le 1$ via

4 $$\begin{aligned} \bar{\alpha } = \dfrac{\ln {(\alpha )} - \ln {(\alpha _{\mathrm {min}})} }{ \ln {(\alpha _{\mathrm {max}})} - \ln {(\alpha _{\mathrm {min}})}}, \;\; \bar{M} = \dfrac{ m - m_{\mathrm {min}} }{m_{\mathrm {max}} - m_{\mathrm {min}}}, \end{aligned}$$

where the subscripts“min” and “max” denote the minimum and maximum value of the parameter that occurs in the dataset.

We train our ANN on $10^4$ samples generated by the low-fidelity model, and at the end of the training phase, the ANN generalizes well (not shown) to the synthetic data that come from the low-fidelity model. However, the ANN trained on reduced physics needs to perform well on synthetic data obtained from the high-fidelity physics model, and its performance is examined in Figs. 7 and 8. When our ANN is trained on data from our reduced-order model and it is asked to predict the true parameters $(\alpha , M)$ that would fit a production curve obtained from a high-fidelity simulation, it predicts say, $(\alpha _1, M_1)$ instead. The first column of Fig. 7 illustrates this discrepancy in true and predicted parameters. When forecasting is done with the parameters $(\alpha _1, M_1)$, the discrepancy in the actual production and forecasted production is depicted in the first column of Fig. 8. The process of transfer learning consists of the ANN training on a handful of such high-fidelity production data with their labelled parameters $(\alpha , M)$. When the ANN after tranfer learning encounters such a production curve, its prediction now becomes $(\alpha _2, M_2)$ (second column of Fig. 7) which is much improved, and the forecasted production curve from the improved prediction $(\alpha _2, M_2)$ is shown in the second column of Fig. 8.

To summarize, in Fig. 7, the parameter prediction from the ANN with parameter loss function (7) before and after transfer learning is compared while in Fig. 8 we compare predicted production profiles obtained with Patzek reduced-order model from the ANN with parameter loss function (7) before and after transfer learning.

Since the ANN is trained to minimize the parameter loss function (7), we observe that it predicts the parameters corresponding to high-fidelity data reasonably. However, those parameters are unable to predict the production accurately, showing that learning from low-fidelity models is not enough, thus demonstrating the need for transfer learning. The figures also showcase the performance on synthetic test data from high-fidelity simulations after transfer learning with 100 high-fidelity runs. We note that the parameter prediction has improved dramatically, and consequently, the production predicted is also very accurate. In these plots, the production predicted by the high-fidelity simulations is assumed to be the ground truth.

We point out that our interest is to illustrate and demonstrate our novel workflow for unconventional reservoirs. Moreover, the goal of real-time decision precludes striving for high accuracy of predicted profiles, since greater accuracy can only come at the expense of computational speed.

The results of the training and transfer learning phase described so far were based on the parameter loss function (7). However, considering that our eventual interest is long-term production, and parameter prediction is only an intermediate step to calculate production, we would like to switch to the production loss function (8) that relates directly to our quantity of interest. With the loss function (8), the results after the training phase with low-fidelity models are shown in Figs. 9 and 10 where, as before we compare the performance on parameter prediction in Fig. 9 and examine the predicted production profiles in Fig. 10.

With the loss function (8), the parameter prediction is no longer the objective, and hence the estimated parameters fare worse than before in Fig. 9. Hence, unlike the improvement seen when comparing the columns of Fig. 7, we do not see any. However, the results of Fig. 10 are similar to the ones encountered earlier in Fig. 8. Thus, the prediction of production remains inaccurate, again showing the need for transfer learning. After transfer learning as before, we see that the predictions of production have improved dramatically in the second column of Fig. 10.

Thus, working with either choice of loss function, we have obtained a machine-learning inverse model (a trained ANN) that is now capable of predicting long-term production once it is supplied with short-term production data.

We would like to emphasize that in the workflow described here, we have supplemented synthetic data from low-fidelity models with synthetic data on long-term production from high-fidelity models. However, one could use site production data if available for re-training during the transfer learning stage. Thus, the flexibility of our proposed workflow makes this approach ideal for unconventionals.

## Conclusion

We presented an alternative, novel workflow for unconventional reservoirs in this article, based on the interplay between reduced-order models and machine-learning. Our PIML workflow addresses the challenges to real-time reservoir management in unconventionals, namely lack of data, need for rapid modeling results during early production, and computational expense of high-fidelity modeling. We used the machine-learning paradigm of transfer learning to bind together fast but less accurate reduced-order models with slow, but accurate high-fidelity models and circumvent the difficulties inherent in the current state-of-the-art for unconventionals. We used the Patzek model as the reduced-order model to generate synthetic production data and supplemented this data with synthetic production data obtained from high-fidelity DFN simulations of the site. Such a PIML workflow, grounded in physics, is a viable candidate for near real-time history matching and production forecasting in a fractured shale gas reservoir. Since the considered model parameters in this study are two scalars, one might wonder if the assumptions could be relaxed to consider spatially varying model parameters. However, spatial model parameters are a complication best avoided because they would necesissate the use of a complex, slow model which would destroy the real-time aspect of our workflow that is needed for operators to find this useful.

The significance of our approach is that while it is developed for MSEEL, it is not wedded to it. We expect the same workflow can be applied to other unconventional hydrocarbon formations (e.g., Woodford, Barnett, Utica, EagleFord) should site data become available, and the same set of ML techniques from transfer learning will be able to model another site/formation. This is a reasonable hypothesis based on the underlying principles of transfer learning. The key to our workflow is to have a physics model that captures the underlying mechanisms to ensure we are doing more than data-fitting. However, we need to make this faster for real-time decisions. Application to another site would require a detailed site model whereas we focussed on making computations faster and defining success if our emulator can mimic our high fidelity model.

Only fine-tuning (or minimal retraining of the neural networks) will be required to transfer knowledge across shale sites/formations and it is a clearly superior alternative to developing a new ML model altogether when considering a different site.

## Data Availability

The datasets generated during and analysed during the current study are available from the corresponding author on reasonable request.
